# Middle-aged and elderly users’ continuous usage intention of health maintenance-oriented WeChat official accounts: empirical study based on a hybrid model in China

**DOI:** 10.1186/s12911-021-01625-4

**Published:** 2021-09-03

**Authors:** Lin Xu, Pengfei Li, Xiaorong Hou, Hongfan Yu, Tingting Tang, Ting Liu, Shoushu Xiang, Xiaoqian Wu, Cheng Huang

**Affiliations:** 1 College of Medical Informatics, Chongqing Medical University, Chongqing, China; 2 College of Public Health and Management, Chongqing Medical University, Chongqing, China; 3 The Children’s Hospital of Chongqing Medical University, Chongqing, China; 4 Medical Data Science Academy, Chongqing Medical University, Chongqing, China

**Keywords:** WeChat official accounts, Health maintenance, Middle aged and elderly, Structural equation model, Continuous usage intention

## Abstract

**Background:**

Although middle-aged and elderly users are the main group targeted by health maintenance-oriented WeChat official accounts (HM-WOAs), few studies have explored the relationship of these accounts and their users. Exploring the factors that influence the continuous adoption of WOAs is helpful to strengthen the health education of middle-aged and elderly individuals.

**Objective:**

We developed a new theoretical model and explored the factors that influence middle-aged and elderly individuals' continuous usage intention for HM-WOA. Performance expectancy mediated the effects of the model in explaining continuous usage intention and introduced health literacy into the model.

**Methods:**

We established a hybrid theoretical model on the basis of the unified theory of acceptance and use of technology 2 model (UTAUT2), the health belief model (BHM), protection motivation theory (PMT), and health literacy. We collected valid responses from 396 middle-aged and elderly users aged ≥ 45 years in China. To verify our hypotheses, we analyzed the data using structural equation modeling.

**Results:**

Performance expectancy (β = 0.383, *P* < 0.001), hedonic motivation (β = 0.502, *P* < 0.001), social influence (β = 0.134, *P* = 0.049), and threat appraisal (β = 0.136, *P* < 0.001) positively influenced middle-aged and elderly users' continuous usage intention. Perceived health threat (β =  − 0.065, *P* = 0.053) did not have a significant effect on continuous usage intention. Both threat appraisal (β = 0.579, *P* < 0.001) and health literacy (β = 0.579, *P* < 0.001) positively affected performance expectancy. Threat appraisal indirectly affected continuous usage intention through performance expectancy mediation.

**Conclusions:**

Our new theoretical model is useful for understanding middle-aged and elderly users' continuous usage intention for HM-WOA. Performance expectancy plays a mediation role between threat appraisal and continuous usage intention, and health literacy positively affects performance expectancy.

## Introduction

### Background

Preventing disease and supporting health self-management have become increasingly important for the growing aging population. Health maintenance information, which plays an important role in safeguarding the physical and mental health of the Chinese public, refers to information that is conductive to strengthening the body's resistance and to intervening with disease [1]. Previous report pointed that middle-aged and elderly users collected health information more frequently than did younger ones [2] and more easily believed in rumors related to health maintenance and food safety [3]. Therefore, the health information behavior and influencing factors of middle-aged and elderly users deserve to be studied.

Compared with traditional methods, social media platforms provide a convenient way for people to obtain health information on mobile phones, thus helping to increase their knowledge without incurring time and space constraints. WeChat is the favorite social platform of Chinese people, about 67% of Chinese people use WeChat [4]. WeChat is also the most popular social platform for middle-aged and elderly users [2]. Similar to Facebook's fan-page, WeChat official account (WOA) is a widely used module on WeChat that can provide different information and services to users with different information needs. Users can subscribe to the WOAs, free access to news and information. Health maintenance-oriented WeChat official accounts (HM-WOAs) are service public accounts that help medical institutions or non-profit organizations and individuals communicate with the society and share health information knowledge such as disease prevention, health care, and disease recovery.

As an important tool to disseminate health information, WOA has been an effective and feasible way to promote health education and improve health literacy [5]. So far, the researches on WOAs mainly focus on the following aspects. The first is a study on the effectiveness of WOA intervention on disease or health behavior. The WOA plays an important role in preventing and treating malaria [6], promoting breastfeeding [7], helping men lose weight [8], supporting the parental care of children with acute lymphoblastic leukemia [9], and preventing suicide among HIV infected and depressed patients [10]. The second is to explore the influence of internal and external characteristics of information on the behavior of using WOAs. The studies found that quantity, content characteristics, organizational structure, multimedia application and article type of health information dissemination affect the information behavior of users [11]. Zhang et al. [12] also explored the factors that influence users to use the official account of China's provincial center for Centers for Disease Control and Prevention. These factors include article content, article type, communication skills, number of marketing elements, article length, and title type.

The reason why users use HM-WOA is that they hope WOA is helpful for their health, but studies show that only 14.4% of users think WeChat health information can improve their health [13]. The HM-WOAs failed to meet users' expectations, which weakened users' intention to continue using it. In fact, WeChat is a good health education tool, it is of great significance to explore the factors that affect the continuous usage intention for HM-WOA. From the current research status, existing studies still focus on the health information itself and the effectiveness of public accounts on health behavior intervention, and few studies have explored the factors affecting the continuous usage intention for HM-WOA.

Continuous usage intention refers to the user's intention to continue using the information system after the initial use of the information system. Bhattacherjee' s regards that although the initial acceptance of an information system is an important first step in achieving the success of an information system, the long-term viability of an information system and its ultimate success depends on its continued use, not its first use [14]. Yuan [15] found that performance expectations, hedonic motivation, price value, and habits are important predictors of users' intention to continue using fitness apps. Zhao [16] explored the factors that affect the willingness of Koreans with physical disabilities to continue using smart devices. Although the continuous usage intention has been widely used in the field of health information systems, there is no research linking it with the HM-WOA.

## Study goal

The purpose of this study is to establish a new model of continuous usage intention and explored the factors that influence middle-aged and elderly individuals' continuous usage intention for HM-WOAs. By reviewing previous studies, we found that the continuous usage intention of middle-aged and elderly users is a comprehensive effect of attitudes, beliefs, knowledge and social environment. Some typical models are used to analyze the intention and behavior of users to continue using health technologies, such as unified theory and technology acceptance model (UTAUT2) [15], health belief model (HBM) [17] and protective motivation theory (PMT) [18]. However, only some of the variables in these typical models are appropriate for our research content and subjects. And to the extent of our knowledge, we have not found any single model applicable to our research. Therefore, we tried to integrate the factors that may affect the continued use of WOAs by middle-aged and elderly users in the three models of UTAUT2, PMT and HBM into a new model. In addition, considering the decline in the physical condition of middle-aged and elderly people and the difference in health, we have added health literacy to the new model. It is worth noting that in previous studies, threat appraisal has been shown to affect performance expectancy, but the mediating role of performance expectancy in threat appraisal and continuous usage intention has not yet been discussed. This research attempts to explore the mediating role of expected performance between them.

### Theoretical basis and research hypotheses

#### Unified theory of acceptance and use of technology 2

UTAUT2 is established on the basis of UTAUT by Venkatesh et al. [19]. The purpose is to explore more comprehensively the factors that influence users to adopt information technology. The UTAUT2 model contains seven factors, which are performance expectancy, effort expectancy, facilitating conditions, and social influence hedonic motivation, price value and habit. The UTAUT2 model has been widely used in research on continuous usage intention in different fields, such as food delivery apps [20] and fitness apps [15].

Performance expectancy is defined as the degree to which individuals perceive that using the system will help them to attain performance. In the context of our study, performance expectancy denotes the belief of middle-aged and elderly users on whether utilizing the HM-WOA has a positive effect on their health. Performance expectancy has a positive relationship with Internet shopping [21] and played an important role in the elderly population's adoption of mHealth and home remote health services [22]. Therefore, the proposed link between performance expectancy and continuous usage intention of WeChat accounts is logical. Thus, we put forward the following hypothesis:

- **H1**: Performance expectancy has a positive impact on the continuous usage intention of HM-WOA.

Social influence reflects beliefs on whether an individual should use a new technology while being affected by their surrounding peer or family group. Social influence has played a critical role in elderly patients' intention to adopt telecare services and precision medicine [23]. Perceived support from families and society has positively affected the health information-sharing behavior among Chinese elderly individuals [24]. We suppose that the support or nonsupport of surrounding people may affect middle-aged and elderly users' utilization of HM-WOA and their continuous usage intention. Therefore, we put forward the following hypothesis:

- **H2:** Social influence has a positive impact on the continuous usage intention of HM-WOA.

Hedonic motivation is defined as the individual's perceived enjoyment from using technology. In the context of our study, hedonic motivation represents the belief that middle-aged and elderly users' utilization of HM-WOA is enjoyable. One study has found that participants from Japan and the United States enjoyed engaging in activities that promote happiness despite their cultural differences in types of daily activities [25]. Similarly, Peng [26] determined that hedonic motivation is an important factor that affects middle-aged and elderly users in the adoption of food safety information from the WeChat public platform. Hence, we put forward the following hypothesis:

- **H3:** Hedonic motivation is positively associated with the continuous usage intention of HM-WOA.

In addition, taking into account the inherent attributes of WOAs and variables, we excluded some variables in UTAUT2. The variables and reasons for exclusion are as follows. First, WOAs provide users with free health information. To some extent, if the cost of adopting health information is lower, the significance level of effort expectancy will also be lower [27], so effort expectancy and price value are excluded from our model. Second, the facilitating conditions in UTAUT model is not a factor that directly affects the prediction intention but a factor that directly affects the prediction behavior, so facilitating conditions is not suitable for our research purpose [28]. Third, UTAUT2 shows that habit can predict behavioral intentions [19], but for the existence of anti-habitual intentions, habit cannot always predict intention [29]. In this study, WeChat is the main source of rumor health information [30], and HM-WOAs fail to help most users improve their health [13]. Low effectiveness of WOAs may make users try to resist their habit of using HM-WOAs. Therefore, this study did not use habit as a factor in predicting the continuous usage intention.

### Health belief model

HBM is a value expectation theory that addresses the desire to avoid disease and the belief that health-related actions can prevent disease [31]. The health model was first used for the cognition and prevention of healthy behaviors. Recently HBM is also commonly applied to explain health behavior intention changes. Educational programs based on HBM and implemented through multimedia can change nutritional concepts and behaviors for the prevention of colorectal cancer [32]. Guidry's investigation [33] found that the HBM model is an important tool for determining the intention of COVID-19 prevention behaviors. Zhang et al. [34] used the health belief model to investigate the willingness of the elderly in rural China to have regular health check-ups.

HBM emphasizes the increasing health attention and adoption of health behaviors when people perceive health threats. Middle-aged and elderly users may perceive more physical-quality health threats than do younger individuals because of their declining physiological conditions. Hence, we focus on the relationship between perceived health threat and continuous usage intention in our research model. According to previous studies, patients who perceive high health threats will more likely adopt smartphone health technology for chronic disease management [35]. Kim and Park [36] also found that patients with perceived high health threats will likely use eHealth services to prevent diseases. Therefore, we put forward the following hypothesis:

- **H4:** Perceived health threat is positively correlated with the continuous usage intention of HM-WOA.

### Protection motivation theory

Understanding the motivation of middle-aged and elderly users to continue to use HM-WOAs is essential to improve the health literacy of them through WeChat. PMT is widely used to analyze people's performance in disease prevention and health promotion. The main factors involved in PMT are threat and coping appraisals [37]. Individuals evaluate the risk or threat before making appropriate behavioral decisions. Previous studies have demonstrated that threat appraisal is an important driver for an individual to engage in health behavior. Deng [38] has determined that threat appraisal is positively correlated with mHealth service adoption. The efficacy of Internet usage has been associated with the reliability of information available on the Internet [39].

In our model, we assume that threat appraisal refers to the assessment of the quality and reliability of information on the WeChat accounts. In addition, the relationship between threat appraisal and continuous usage intention of online health consultation services has been positively mediated by the perceived benefit [40]. Thus, we put forward the following hypotheses:

- **H5:** Threat appraisal is positively associated with the continuous usage intention of HM-WOA.
- **H6:** Threat appraisal is positively associated with performance expectancy.
- **Ha:** Performance expectancy plays a mediating role in threat appraisal and continuous usage intention of HM-WOA.

Coping appraisal refers response efficiency, self-efficacy, and response cost. Response efficiency refers to the utility of the HM-WOA and its meaning is similar to the expected performance. In addition, Like the reasons for effort expectancy and price value exclusion, self-efficacy, and response cost are not suitable for our research. Therefore, in this study, only part of the meaning of the coping appraisal is consistent with our research purpose, and this part of the meaning is reflected in the performance expectancy.

### Health literacy

Given that health literacy describes skills and abilities that enable people to obtain, understand, and apply health information, it can have a positive impact on health [41]. Health literacy can affect a person's ability to judge health information and their processing of that information [42]. Health literacy also has been typically considered in the fields of health care [43], promotion [44], and education [45] in this era of an explosion in available Internet health information [46]. Chen et al. found that people with lower levels of health literacy are more likely to trust and adopt health information from social media, even if such data include pseudoscientific health details, whereas individuals with higher health literacy can better understand health information, thereby increasing their expected application of this information. Similarly, Kate [47] has shown that performance expectancy is an important determinant to account for eHealth. Hence, we put forward the following hypothesis:

- **H7:** Health literacy is positively associated with performance expectancy of HM-WOA.

### Modified research model

**Fig. 1:**
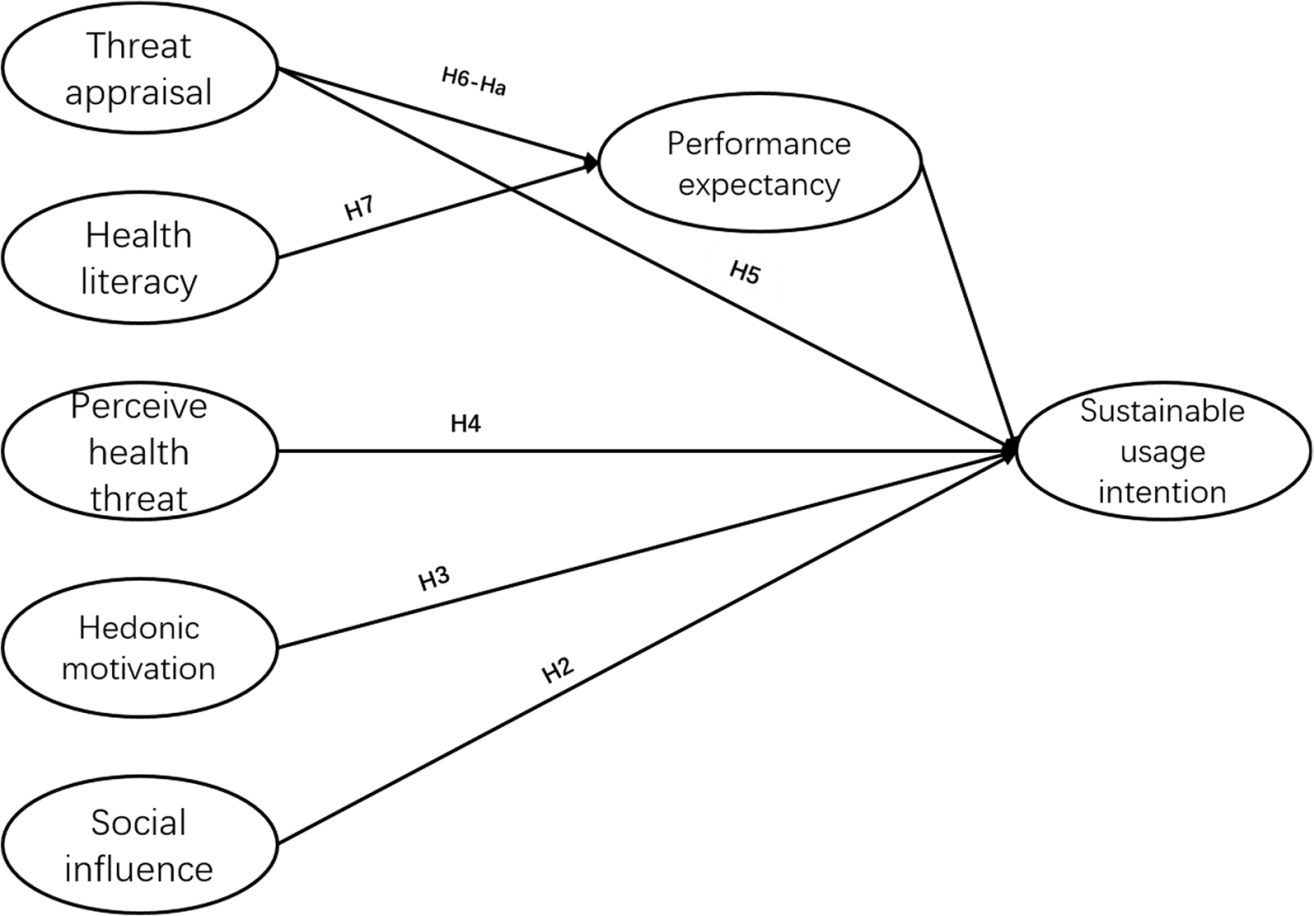
Research model

Based on the above theories and hypotheses, our new model attempts to explore 6 factors that affect the continuous usage intention of WOAs by middle-aged and elderly users. Figure 1 shows the relationship between the six factors and the continuous usage intention. Notably, different from previous research models, health literacy and threat appraisal are used as predictors of expected performance, and consider performance expectancy a mediation between threat appraisal and continuous usage intention.

## Methods

### Measurements

All items in this research model referred to in previous studies were repeatedly revised to validate the aforementioned hypotheses, to match our research content, and to improve clarity. performance expectancy applies three items adapted from Lin [48], Deng [49], and Koivumäki et al. [50]. SI was measured using four items tested by Wang [51]. Hedonic motivation was verified using four items from Peng et al. [26]. Perceived health threat included three items adapted from Deng [49] and Lee et al. [52]. Threat appraisal was verified using three items demonstrated by Rieh [53] and Huang [54]. Health literacy includes three items introduced by Abel [55] and Su et al. [56]. Finally, continuous usage intention includes three items used by Venkatesh [57] and Bhattacherjee [58].

### Questionnaire design

Our questionnaire consists of two parts related to demographic and variable factors. According to the age classification of Pubmed and Mesh, the research subjects were divided into three age groups and 45 years old was set as the minimum standard for middle-aged and elderly [59, 60]. We measured items of predicted variables using a 5-point Likert scale ranging from *strong disagreement* (1) to *strong agreement* (5). We designed two questions to assess the truth in the respondent's answer to ensure the questionnaire's validity. The question “Have you ever used an official WeChat account related to health maintenance?” was designed as a confirmatory question. Moreover, “aged under 45” was set as the confirmatory answer to question “How old are you?” to prevent WeChat users younger than middle age from mistakenly answering the questionnaire. The modified questionnaire obtained satisfactory internal consistency (Cronbach's alpha = 0.954).

### Data collection

We conducted this investigation by adopting a combination of online and offline modes. This was done to expand the sample size and enhance the representativeness of the sample. We used QQ, WeChat and other social platforms to obtain data through forwarding by classmates, friends, etc. We limited the IP addresses of the online participants to prevent a single user from filling in the questionnaire more than once. At the same time, we used random interception to obtain offline data in places where there are more middle-aged and elderly people, such as the Olympic Sports Center, supermarkets and parks. All respondents voluntarily participated in this survey. All research protocols in this study were approved by the Medical Research Ethics Committee of Chongqing Medical University on September 15, 2020.

A total of 416 questionnaires were collected. A total of 396 valid questionnaires were retained, with an effective rate of 95%. The effective sample size, which is more than 10 times the measuring items, meets the requirement of sample stability. We used the following inclusion criteria to screen target participants: (1) the participants were aged ≥ 45 years; (2) participants owned smartphones, which were used to access the HM-WOA; and (3) the participants were literate and had no communication barriers.

### Data analysis

We used frequency and percentages to analyze the participants' general characteristics. We evaluated reliability and validity of the questionnaire using IBM SPSS version 24.0. We used structural equation modeling (SEM) to assess the research model. SEM is a multivariate statistical method that integrates two statistical methods: factor and path analyses. The data analysis was conducted in two stages by using IBM SPSS AMOS version 21.0.

In the first stage, we used confirmatory factor analysis to assess the measurement items' reliability and validity. We used correlation analysis to test the strength of the relationship between two variables. We tested common method bias (CMB) using a correlation matrix.

In the second stage, we examined the structural model and tested the stated hypotheses. We used the bootstrap method for repeated sampling, with the value set to 1000 and confidence intervals set as 95%, and then we conducted the empirical test of the model so as to avoid problems such as the deviation of operation results caused by the non-normal distribution of the sample data. Thus, a small sample estimating a large sample can be realized. The significance level for all hypotheses was alpha < 0.05.

## Results

### Demographic description

The description statistics revealed a valid sample of 36.4% men and 63.6% women in our survey. The majority of participants were aged 45–64 years (76.3%, *n* = 302), followed by 65–80 years (21.7%, *n* = 86), and > 80 years (2%, *n* = 8). The majority of the respondents (79.8% [316/396]) had an average monthly income of < CN¥5000. Only a few respondents had an average monthly income > CN¥10,000 (3.5% [14/396]). Furthermore, most of the respondents graduated from junior high school (34.6%, *n* = 137). Unexpectedly, approximately 50% (198/396) had attended high school. The results are listed in Table 1.

**Table 1:** Demographics of the questionnaire respondents (N = 396)

Demographic	N	Percentage
*Gender*		
Male	144	36.4
Female	252	63.6
*Age group*		
45–64	302	76.3
65–79	86	21.7
≥ 80	8	2.0
*Education*		
Elementary school and below	61	15.4
Junior high school	137	34.6
Senior high school	74	18.7
College	48	12.1
Undergraduates	69	17.4
Master	6	1.5
Doctor	1	0.3
*Income*		
< 3000	146	36.9
3001–5000	170	42.9
5001–10,000	66	16.7
> 10,000	14	3.5

### Measurement model

We used Cronbach's alpha and composite reliability (CR) to test the reliability of the measurement model in this study. Cronbach's alpha and CR greater than 0.7 indicated satisfactory construct reliability and internal consistency of the measurement model [61]. This study demonstrates acceptable content validity because most of these measurement items were revised using the measurement items of previous studies. We tested convergent validity of the model by verifying average variance extracted (AVE) and standardized factor loading. AVE values of all potential factors > 0.5 and standardized factor loadings of all items > 0.6 confirm the acceptable convergent validity of the measurement model [62]. The square root of AVE of each construct can be used to assess discriminant validity if the value is significantly greater than the correlation of any two constructs, thereby indicating the model's acceptable discriminant validity [63]. The results are listed in Table 2.

**Table 2:** Construct reliability and composite reliability

Construct	Item	Factor loading	Cronbach's alpha	CR	AVE
PE	PE1	0.939	0.908	0.855	0.774
	PE2	0.890			
	PE3	0.805			
SI	SI1	0.822	0.865	0.838	0.565
	SI2	0.888			
	SI3	0.703			
	SI4	0.733			
PHT	PHT1	0.814	0.860	0.855	0.774
	PHT2	0.818			
	PHT3	0.827			
TA	TA1	0.816	0.892	0.855	0.774
	TA2	0.915			
	TA3	0.841			
HM	HM1	0.839	0.901	0.838	0.565
	HM2	0.871			
	HM3	0.853			
	HM4	0.780			
HL	HL1	0.730	0.778	0.855	0.704
	HL2	0.865			
	HL3	0.616			
BI	BI1	0.912	0.918	0.918	0.789
	BI2	0.875			
	BI3	0.877			

We examined the CMB via the correlation matrix of constructs. If any correlation is higher than 0.90, then the research data likely suffer from CMB [57]. As shown in Table 3, the highest correlation of 0.814 suggests that CMB was not a serious problem in this study.

**Table 3:** Correlations and discriminant validity

	AVE	PHT	BI	TA	PE	HM	HL	SI
PHT	0.774	0.880^a^						
BI	0.789	0.183	0.888^a^					
TA	0.774	0.327	0.651	0.880^a^				
PE	0.774	0.223	0.795	0.623	0.880^a^			
HM	0.565	0.244	0.814	0.608	0.724	0.752^a^		
HL	0.704	0.133	0.431	0.134	0.351	0.503	0.839^a^	
SI	0.565	0.235	0.757	0.588	0.784	0.760	0.450	0.752^a^

^a^Square root of average variance extracted

### Structural model

The results of the structural model's fitness index are shown in Table 4. Chi-squared/degree of freedom (χ^2^/df) = 2.719, root mean square error of approximation = 0.066, standardized RMR = 0.062, goodness-of-fit index (GFI) = 0.892, adjusted GFI = 0.859, comparative fit index (CFI) = 0.944, normed fit index (NFI) = 0.914, and incremental fix index (IFI) = 0.944 indicate the acceptable fit of our structural model.

**Table 4:** The fitness index of the structural model

Mode-fit index	Recommended value	Scores	Comment
Χ^2^/df	≤ 3	2.719	Ideal
GFI	≥ 0.8	0.892	Acceptable
AGFI	≥ 0.8	0.860	Acceptable
CFI	≥ 0.8	0.944	Ideal
NFI	≥ 0.8	0.914	ideal
IFI	≥ 0.8	0.944	Ideal
SRMR	≤ 0.08	0.062	Acceptable
RMSER	≤ 0.08	0.066	Acceptable

The results of structural parameter estimate and hypotheses are listed in Table 5. Performance expectancy (β = 0.383, *P* < 0.001) had a positive effect on the continuous usage intention of HM-WOA. Hence, Hypothesis 1 is acceptable. Social influence (β = 0.134, *P* = 0.049), hedonic motivation (β = 0.502, *P* < 0.001), and threat appraisal (β = 0.136, *P* < 0.001) had a significant positive effect on the continuous usage intention of HM-WOA. Therefore, Hypotheses 2, 3, and 5 are valid.

**Table 5:** Structural parameter estimates

Hypothesized path	Standardized path coefficients	S.E.	C.R.	*P*
H1:PE → BI	0.383	0.052	7.298	< 0.001
H2:SI → BI	0.134	0.068	1.964	0.049
H3:HM → BI	0.502	0.071	7.053	< 0.001
H4:PHT → BI	− 0.065	0.033	− 1.938	0.05
H5:TA → BI	0.136	0.054	2.507	0.01
H6:TA → PE	0.579	0.044	13.073	< 0.001
H7:HL → PE	0.426	0.057	7.423	< 0.001

However, perceived health threat (β =  − 0.065, *P* = 0.053) was insignificantly correlated with continuous usage intention. Thus, Hypothesis 4 is rejected.

In addition, threat appraisal positively affected performance expectancy (β = 0.579, *P* < 0.001), and health literacy has a significant relationship with performance expectancy (β = 0.426, *P* < 0.001). Hence, Hypotheses 6 and 7 are acceptable.

### Mediation results

Threat appraisal has a significant influence on continuous usage intention in terms of its mediating effect. Our research model demonstrated the significant indirect effect of threat appraisal on continuous usage intention through performance expectancy (β = 0.222, *Z* > 1.96), thereby indicating the existence of the significant mediating effect of performance expectancy between threat appraisal and continuous usage intention. Hence, Hypothesis a is acceptable (Table 6).

**Table 6:** Mediation results

TA → PE → BI	Point estimate	Product of coefficients		Bootstrapping 95% CI			
				Bias-corrected		Percentile	
		SE	Z	Lower	Upper	Lower	Upper
Indirect effect	0.222	0.061	3.639	0.1	0.344	0.109	0.346
Direct effect	0.136	0.062	2.194	0.026	0.277	0.026	0.277
Total effect	0.357	0.067	5.328	0.236	0.501	0.242	0.504

## Discussion

### Principal findings

Consistent with Hypothesis 1, performance expectancy has a positive effect on the continuous usage intention. This result shows that when users perceive that the HM-WOA is more helpful to their health, their intention to continue using the HM-WOA is stronger. This finding is supported by previous study, performance expectancy is a determinant of the intention of the elderly in the community to use e-health [64]. In addition, our results confirmed Hypothesis 7, indicating that the higher the health literacy of middle-aged and elderly users, the higher the performance expectations of WeChat health maintenance official accounts. Hennemann [65] suggested that e-health literacy can indirectly affect occupational e-mental health acceptance intentions through expected performance.

Our results suggest that social influence has a positive effect on the continuous usage intention of HM-WOA. Thus, Hypothesis 2 was supported. Similar to the results of previous studies, family and friends can influence the intention to use mobile health technology, and a significant correlation exists between social influence and continuous usage intention [23, 66]. Liu et al. [24] also found that social influence positively affected the elderly' s sharing behavior of health information in China. In keeping with Hypothesis 3, hedonic motivation has a positive effect on the continuous usage intention for the middle-aged and elderly individuals. Previous studies have found that hedonic motivation is one of the factors that promote the adoption of food safety information on WOAs by middle-aged and older users. Meanwhile, Ramirez-Correa et al. [67] also found that perceived enjoyment can enhance the willingness of the elderly to use SNS.

Contrary to our aforementioned Hypothesis 4, a significant relationship is absent between perceived health threat and continuous usage intention. However, Dou et al. [68] found that perceived health threats enhance users' intention to use hypertension monitoring apps. Inconsistent results may be due to different characteristics of research platforms. The hypertension monitoring app can provide users with reliable hypertension monitoring reports, but the quality of the information on the health WOA is difficult to guarantee. When patients encounter more serious diseases, they are more willing to trust doctors who can provide them with accurate diagnosis and treatment information than WOAs. This view also was supported by Benedicta's study [69], only when parents thought that their child's condition was not serious did they access online health information to manage their child's health.

In our model, threat appraisal refers to the assessment of the quality and reliability of information on the WOAs. Consistent with our Hypothesis 5 and Hypotheses 6, this study confirms the threat appraisal has a positive effect on continuous usage intention and performance expectancy. Jin et al. found information quality and source credibility have a significant positive impact on the adoption possibility of healthcare information [70]. Meanwhile, what makes us happy is, Hypothesis a is supported. Our study demonstrated mediating effects of performance expectancy and tested the significant association between threat appraisal and continuous usage intention through performance expectancy.

## Theoretical implications

Our research has important theoretical significance. First, the results of this study can provide a new reference for continued use intention research. Although many scholars have conducted research on the continuous usage intention of different health technologies and put forward corresponding models. However, few studies explored the mediating effect of expected performance. This study found that expected performance had a significant mediating effect between threat appraisal and continuous usage intention.

Second, the current researches on WOA mainly focus on health information itself and the effectiveness of interventions in health behaviors. Few studies linked the WOA to continuous usage intention. To bridge this gap, this study took middle-aged and elderly users as the research object and explored the factors that affect their continuous usage intention for HM-WOA.

Third, with the intensification of the aging process and the rapid development of the mobile Internet, the willingness of middle-aged and elderly users to use online health information technology should be paid attention to. However, there are currently few studies on the continued use of online health technologies by middle-aged and elderly users. We hope that this study can provide a reference for future research on online health behavior intentions of middle-aged and elderly users.

## Practical implications

Our study also presented several practical implications. The results of this study can provide a certain reference for HM-WOAs manager to improve users' continuous usage intention. First of all, health literacy has a positive impact on expected performance. and expected performance has an obvious mediating effect between threat appraisal and continuous usage intention. In order to meet the user's expected performance, attract users to continue to use HM-WOA. HM-WOAs managers should strengthen the health information review mechanism to ensure the scientificity and reliability of the information. At the same time, accurate identification of HM-WOA users with different health literacy should be realized and a personalized information push mechanism should be developed.

Secondly, social influence has a positive effect on the continuous usage intention, so we suggest that HM-WOAs can invite celebrities to increase users' intention to continue use through their social influence. In addition, hedonic motivation is a positive predictor of continuous usage intention. The HM-WOA managers can set up small games or read rewards. This method not only satisfies the user's entertainment, but also stimulates the user to repeatedly use the healthy HM-WOAs.

## Limitations

The 396 sample number is too small to represent the middle-aged and elderly users who used HM-WOA. Fortunately, the number is greater than 10 times the measuring items and meets the sample requirements of SEM. Thus, our sample was representative to a certain degree.

The scope of this study is limited to the survey conducted for middle-aged and elderly users rather than to all age groups. Future studies can focus on using other age groups to examine the effects of age on continuous usage intention. In addition, although we grouped gender, age, education and income, we did not explore the differences among these variables. We plan to study these variables in future research when we obtain more valid samples.

Furthermore, we considered only fundamental health literacy and ignored other types, such as interactive and media health literacy. Future studies are recommended to include other types of health literacy and determine their effects on continuous usage intention of HM-WOA. Finally, the original innovation of health information in WOAs may also affect the intention of continuous use, and we may take the original innovation as a moderating variable into our model in a future study.

## Conclusions

An integrated model of UTAUT2, HBM, PMT, and health literacy developed in this study explored the factors that affect middle-aged and elderly users' continuous usage intention of HM-WOA. The results show that continuous usage intention is positively associated with performance expectancy, threat appraisal, and hedonic motivation but negatively associated with perceived health threat. Meanwhile, performance expectancy has a positive mediating effect between health literacy and continuous usage intention. Performance expectancy is also a mediating factor between threat appraisal and continuous usage intention.

These findings are an important extension of the results of previous research models and provide a theoretical basis for health information behavior on the Internet. Our research has certain referential significance for health information publishers and the administrator of HM-WOA.

## Data Availability

The empirical research datasets can be obtained from the corresponding author on reasonable request.

## Abbreviations

AVE: Average variance extracted
CFI: Comparative fit index
CMB: Common method bias
CR: Composite reliability
GFI: Goodness-of-fit index
HBM: Health belief model
HL: Health literacy
HM: Hedonic motivation
IFI: Incremental fix index
NFI: Normed fit index
PE: Performance expectancy
PHT: Perceived health threat
PMT: Protection motivation theory
SEM: Structural equation modeling
SI: Social influence
TA: Threat appraisal
UTAUT: Unified theory of acceptance and use of technology
UTAUT2: Unified theory of acceptance and use of technology 2
WOA: WeChat official account
HM-WOA: Health maintenance-oriented WeChat official account
